# Long term highly saturated fat diet does not induce NASH in Wistar rats

**DOI:** 10.1186/1743-7075-4-4

**Published:** 2007-02-21

**Authors:** Caroline Romestaing, Marie-Astrid Piquet, Elodie Bedu, Vincent Rouleau, Marianne Dautresme, Isabelle Hourmand-Ollivier, Céline Filippi, Claude Duchamp, Brigitte Sibille

**Affiliations:** 1 Laboratoire de Physiologie Intégrative, Cellulaire et Moléculaire, CNRS, Université Lyon 1, F-69622 France; 2 Imagerie Fonctionnelle et Métabolique en Oncologie, EA 3916, Département de Nutrition et d'hépatogastroentérologie, CHU Côte de nacre, Caen, F-14033 France; 3 Department of Hepatology, Chancellor's Building, 49 Little France Crescent, Edinburgh, UK

## Abstract

**Background:**

Understanding of nonalcoholic steatohepatitis (NASH) is hampered by the lack of a suitable model. Our aim was to investigate whether long term high saturated-fat feeding would induce NASH in rats.

**Methods:**

21 day-old rats fed high fat diets for 14 weeks, with either coconut oil or butter, and were compared with rats feeding a standard diet or a methionine choline-deficient (MCD) diet, a non physiological model of NASH.

**Results:**

MCDD fed rats rapidly lost weight and showed NASH features. Rats fed coconut (86% of saturated fatty acid) or butter (51% of saturated fatty acid) had an increased caloric intake (+143% and +30%). At the end of the study period, total lipid ingestion in term of percentage of energy intake was higher in both coconut (45%) and butter (42%) groups than in the standard (7%) diet group. No change in body mass was observed as compared with standard rats at the end of the experiment. However, high fat fed rats were fattier with enlarged white and brown adipose tissue (BAT) depots, but they showed no liver steatosis and no difference in triglyceride content in hepatocytes, as compared with standard rats. Absence of hepatic lipid accumulation with high fat diets was not related to a higher lipid oxidation by isolated hepatocytes (unchanged ketogenesis and oxygen consumption) or hepatic mitochondrial respiration but was rather associated with a rise in BAT uncoupling protein UCP1 (+25–28% vs standard).

**Conclusion:**

Long term high saturated fat feeding led to increased "peripheral" fat storage and BAT thermogenesis but did not induce hepatic steatosis and NASH.

## Background

Non-alcoholic fatty liver diseases (NAFLD) are characterized by triglyceride accumulation in hepatocytes (i.e., liver steatosis). In some cases, steatosis becomes complicated by inflammation and can evolve to apoptosis, necrosis and fibrosis. This association of steatosis to other lesions is called non-alcoholic steatohepatitis or NASH [1], and may evolve into cirrhosis and hepatocellular carcinoma.

NASH is a disease of emerging importance and is now considered as the most common cause of chronic liver disease in the USA [2,3]. While the pathogenesis of NASH is poorly understood, the hypothesis of two "hits" is recognized [4]. Fat accumulation in the liver represents the "first hit". The factor responsible for the second "hit" is hepatic oxidative stress due to ROS emission and/or increased cytokine release, enhancing lipid peroxidation, mitochondrial DNA and respiratory chain damages [5,6]. Currently, no defined therapy is known to alter the course of NASH [5,7-10].

The study of the pathogenic factor involved in NASH is difficult because of the lack of a suitable experimental animal model [11]. Currently, available animal models are rodents either with a genetic defect (ob/ob mice or Fa/Fa rat) [12] or fed a methionine and choline deficient diet (MCD diet) [13]. The latter model is commonly used but induces a nutritional deficiency that is not observed in patients with NASH. The major disadvantage of these models is that they fail to reflect the multi-factorial features of NASH observed in patients. High caloric intake and obesity are factors frequently associated with NASH in humans. In rodents, however, the situation is less clear as rats fed high fat diets were shown to develop hepatic steatosis in some studies [14-16] but not in others [17,18]. Comparisons of the protocols used showed that the composition and the palatability of the diets may play an important role in the development of the obesity and NASH. To overcome these difficulties, some authors gave diet ad libitum while others strictly controlled the caloric intake through intragastric diet infusion or force-feeding. Lieber et al. used a liquid high fat diet given ad libitum to rats [19] whereas Zou et al. controlled daily fat intake by force-feeding rats [20]. In these two cases, high fat diet induced mild steatosis (two fold increase in hepatic triacylglycerol compared to control) and huge hepatic inflammation. The main fat component of these two diets was corn oil, (consisted of 13% (w/w) saturated fatty acid (SFA), 24% monounsaturated fatty acid (MUFA) and 59% polyunsaturated fatty acid (PUFA)). These PUFA were almost entirely composed by pro-inflammatory n-6 polyunsaturated fatty acids which are known to be involved in liver oxidative stress [21]. These models do not really mimic human NASH diet features since a study reported that patients with NASH usually have a diet with higher levels of SFA (13.7% instead of 10.0% total kcal) and cholesterol, and low levels of PUFA (3.5% (w/w) [22].

Consequently, to analyse NASH pathogenesis, the aims of the present study were (i) to study the development of steatosis following a SFA-rich diet, ii) to study the possible evolution from steatosis to NASH and iii) to determine the possible liver adaptations to this new condition. We tested two high saturated fat diets with either coconut oil, which contains roughly 86% SFA, or butter, which contains 51% SFA. These diets were compared with the MCD diet, the most common diet used to mimic NASH in rodents.

## Methods

### Animals and diets

Our study was performed following the recommendations provided by the European Convention for the protection of Vertebrate Animals used for Experimental and Scientific purposes (Council of Europe N° 123, Strasbourg, 1985). Male Wistar rats, purchased from Charles River Laboratories France, were housed four per box on a 12-h light/dark cycle. Animals were allowed to acclimatise to their new conditions for a week before the study. At the age of 21 days the animals were allocated to four different groups. The first group received a standard diet (A04 – Scientific Animal Food & Engineering, France) (std, n = 10). The second group had the choice between standard diet and coconut diet (coco, n = 10), for 14 weeks. The third group had the choice between standard diet and butter diet (butter, n = 4) for 14 weeks as well. The methionine and choline deficient diet (MCDD n = 10; ICN Pharmaceuticals France SA, ref n° 960439), was given for 6 weeks from the age of 9 weeks. Diets components are shown in Table 1. The coconut or butter diets had 67% of energy derived from fat compared with the standard diet (7%). High fat diets were weekly prepared in our laboratory in pellets and stored at +4°C. The food was renewed every day during the treatment and the standard and high fat diet intakes are monitored daily independently. Rats had free access to food and water and were weighed every week for the duration of the study.

**Table 1:** Components (g/kg) of the standard, coconut, butter and methionine-choline deficient (MCD) diets and their fatty acid (FA) composition.

	**standard diet**	**coconut diet**	**butter diet**	**MCD diet**
**protein**	**160**	**170**	**170**	175*
**fat**	**27**	**460**	**460**	**100**
cholesterol	-	-	0.9	-
saturated FA	3	398	236	15
monounsaturated FA	8	27	97	30
polyunsaturated FA	14.5	8	14	55
ω6	14.5	8	12	54
ω3	traces	-	2	1
**carbohydrate**	**596**	**250**	**250**	**655**
**cellulose**	**44**	**20**	**20**	**30**
**mineral**	**50**	**50**	**50**	**40**
**moisture**	**123**	**50**	**50**	

(*: expressed as amino acids)

### Histological study

Histological analyses were achieved in the gastroenterology service of Caen hospital (France). Liver specimens were fixed in 4% buffered formalin for 24 to 48 h. They were then embedded in paraffin, cut at 5 μm, and routinely stained with hematoxylin-eosin (H&E) and reticulin. Severity of steatosis was evaluatedusing the percentage of macrovesicular fat within hepatocytes: mild (5 to 30 %); moderate (30 to 60 %); severe (more than 60%) [23].

### Mitochondrial preparation and utilisation

The liver was rapidly removed and finely minced and washed with ice-cold isolation medium containing 250 mM sucrose, 2 mM KH_2_PO_4_, 1 mM EGTA and 20 mM Tris-HCl (pH 7.2). Liver mitochondria were prepared by standard differential centrifugation procedures [24]. Mitochondrial protein content was determined by the Biuret method [25] with serum albumin as standard. For the determination of oxygen consumption, mitochondria were incubated at a concentration of 2 mg/mL in an oxygraph vessel with a Clark electrode, thermostatically controlled at 37°C, in a medium containing 125 mM KCl, 1 mM EGTA, 2 mM KH_2_PO_4_, 20 mM Tris-HCl with 0.1% fatty acid-free Bovin Serum Albumin (pH 7.2). The control state of respiration was initiated by the addition of 5 mM succinate/0.5 mM malate, in the presence of rotenone (1.25 μM) while the addition of 1 mM ADP initiated the active state of respiration (state 3). Oligomycin (1.25 μg/mg protein) was then added to the mitochondrial suspension to determine the non-phosphorylating respiratory rate (state 4).

### Hepatocyte isolation and closed vials incubation

Hepatocytes were isolated from 20–24 h starved rats as previously described by Berry and Friend (Berry, 1969) and modified by Groen et al. [26], by a two-step in situ collagenase perfusion technique. Hepatocytes (10 mg/mL dry weight) were incubated at 37°C in closed vials containing a Krebs-bicarbonate buffer (120 mM NaCl, 4.8 mM KCl, 1.2 mM KH_2_PO_4_, 1.2 mM MgSO_4_, 24 mM NaHCO_3_, pH 7.4) saturated with 95% O_2_/5% CO_2 _containing BSA (2%, w/v) and 2.4 mM CaCl_2_. Experiments were performed with 20 mM dihydroxyacetone, with or without fatty acids, 4 mM octanoate or 2 mM oleate. After 20 min, oxygen uptake (*J*O_2_) was measured polarographically at 37°C with a Clark electrode before and after the addition of 6 μg/ml oligomycin. At t = 0 and 30 min, samples of hepatocyte suspension were taken, quenched in HClO_4 _(4% v/v final concentration) and neutralized with 2 M KOH/0.3 M MOPS for later enzymatic measure of 3-hydroxybutyrate, acetoacetate and glucose as described by Bergmeyer [27].

### Adipocyte isolation

Adipocytes were prepared from retroperitoneal white fat pads. 1.5 g of fat tissue was digested for 30 min at 37°C with 2 mg/mL of type II collagenase. The digestion medium was a Krebs-Ringer medium (139 mM NaCl, 5.4 mM KCl, 1 mM NaH_2_PO_4_, 1 mM MgSO_4_, 2.2 mM CaCl_2_, pH 7.4) buffered with 20 mM Hepes, containing 2% (w/v) BSA with 7 mM glucose. Isolated cells were obtained by filtration through a coarse nylon mesh (250 μm) before being washed twice with a 2% BSA buffer. Adipocytes were then observed under a microscope and the determination of the diameter was measured on pictures of roughly 200–250 cells using a calibrated scale.

### Biochemical analyses

Liver triacylglycerol concentration was estimated from glycerol release after ethanolic KOH hydrolysis [28], using a commercial colorimetric kit (Biomerieux, France).

### Western blot analysis

Mitochondria from brown adipose tissue (BAT) were prepared as described previously. 40 μg of BAT mitochondrial proteins were separated by SDS-PAGE (12.8% acrylamide) and transferred to polyvinylidene fluoride membranes (Immobilon-P, Millipore). Immunological detection was performed using a rabbit antiserum against UCP1 (α-diagnostics UCP11-S (1:15000), USA). The detection was realized with a horseradish peroxidase-coupled anti-rabbit (Bio-Rad 170–6516 (1:5000)) secondary antibody and an enhanced chemiluminescence (ECL) detection kit (Amersham, UK). Quantification of autoradiographs was performed by scanning densitometry.

### Enzyme assays

BAT samples (40–50 mg) were immediately homogenized at +4°C in 0.3 M phosphate buffer containing 0.05% bovine serum albumin (pH 7.7) using a glass Potter-Elvehjem homogenizer. Then, they were frozen at -80°C and thawed three times to disrupt the mitochondrial membrane. 3-hydroxyacyl-CoA dehydrogenase (HAD, EC 1.1.1.35) was spectrophotometrically determined as previously described by Lowry & Passonneau [29]. Enzyme activity was determined at 25°C and expressed as micromoles of substrate per minute per milligram of protein.

### Statistical analysis

Differences between groups were determined using non-parametric Mann & Whitney tests. Data were expressed as mean values ± SEM and differences between means were considered significant when P < 0.05.

## Results

### Caloric intake, body and tissue mass

**Figure 1:**
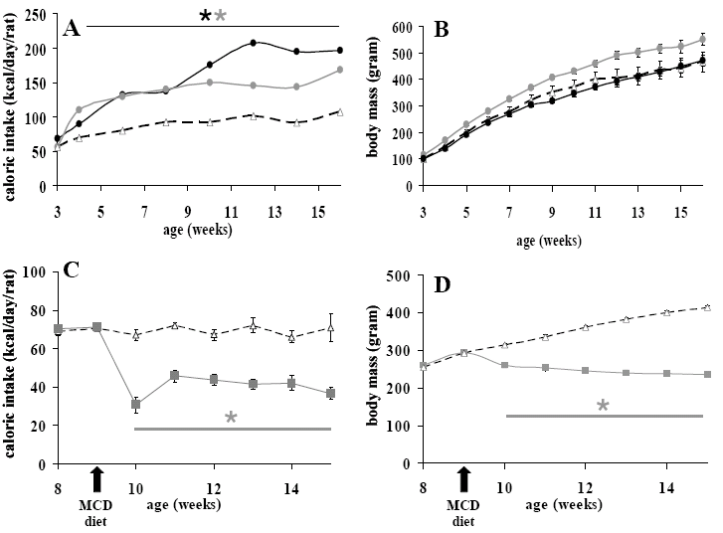
Estimated daily caloric intake (A and C) and body mass (B and D) in different diet groups over time. Rats received either a standard diet (white triangles), a coconut diet (black circles), a butter diet (grey circles) or a methionine-choline deficient diet (grey squares). Values are mean ± SEM from 4 to 10 animals per group, *P *< 0.05:  different from standard.

**Figure 2:**
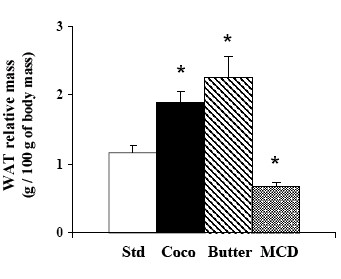
Relative weight of retroperitoneal white adipose tissue (WAT) in rats fed either a standard (Std), coconut (Coco), butter (Butter) or methionine-choline deficient diet (MCD). Data are means ± SEM; n = 10 for standard, coco and MCD groups, and n = 4 for butter group. *P *< 0.01 vs standard diet.

**Figure 3:**
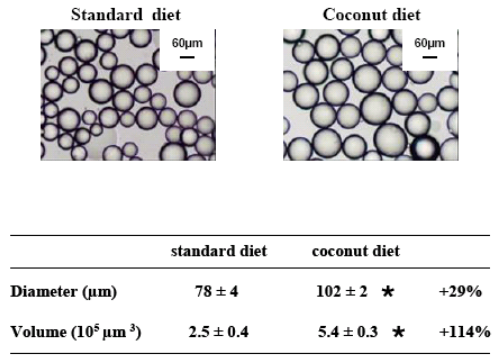
Diameter and volume of isolated adipocytes from the retroperitoneal white adipose tissue of rats fed standard diet or coconut diet. Measures were realised in triplicate on 3 rats per group; values are means ± SEM, *P *< 0.001 vs standard diet.

The rats received either a standard diet with 7% of energy derived from fat, or had free choice between the standard diet and high fat diets with 67% of energy derived from fat. The first high fat diet contained coconut oil, composed of 86% of saturated fatty acid and especially lauric acid (C12:0). The second high fat diet was realised with milk butter that contains 51% of SFA, the most abundant being palmitic acid (C16:0). The respective composition of the two diets in the main fatty acids is presented in Table 2. Because the rats had free access to either standard (2900 kcal/kg) or high fat diets (5500 kcal/kg) we first estimated total calorie ingestion per day and monitored resulting body mass. Animals were housed four per cage. Rats could freely choose between the standard and the high fat diet which were both available within the cages. The amount of food consumed per day from each respective diet was calculated and then that amount was divided by four to estimate the consumption of each rat. As shown in Fig. 1A, the total caloric intake was higher in both groups fed with high fat diets than in rats fed the standard diet. These caloric intakes at the end of the experiment were 143% higher for coconut group (178 kcal/day/rat) and 30% higher for butter group (95 kcal/day/rat), as compared with standard group (73 kcal/day/rat). The profile of lipids, proteins and carbohydrates ingestion at weeks 3 and 16 of experiment is reported in Table 3 for each group. The proportion of energy derived from lipids was increased 1.5 fold for the coconut group to reach 45% by the end of the protocol, while it reached 42% at week 16 of diet in the butter group. At the end of the study period, total lipid ingestion, expressed as a percentage of the calories ingested, was higher in both high fat diet groups than in the standard diet (coconut 45% vs butter 42% vs standard 7% kcal _total_). In the coconut group, this energy came mostly from SFA (41.3%) whereas in the butter group energy was shared between SFA and MUFA (28% and 12% respectively) (Table 3). Surprisingly, in spite of a larger energy intake, body mass was not affected in rats fed the high fat diets (Fig. 1B). However, fat mass was largely affected. Hence, retroperitoneal white adipose tissue depot (Fig. 2) was heavier in high fat diet rats (+62% in coconut and +93% in butter) than in standard rats. To investigate which factors were implicated in this fat modification, adipocytes were isolated from retroperitoneal white adipose tissue, from coconut and standard groups and their size and volume measured. A two-fold increase in cell volume was observed with the coconut diet, demonstrating that the change in white adipose tissue mass was partly due to a change in the volume of individual adipocytes (Fig. 3). Hence, the high fat diet groups displayed the expected feature related to high lipid ingestion, i.e. retroperitoneal fat accumulation and adipocyte growth.

**Table 2:** Percentage of the main fatty acid (FA) measured in the coconut oil and butter.

	**%**	**Coconut oil**	**butter**
**Saturated FA**		**86.5**	**51.3**
	10:0	6	2.5
	12:0	44.6	2.5
	14:0	16.8	7.4
	16:0	8.2	21.7
	18:0	2.8	10
**Monounsaturated FA**		**5.8**	**21**
	18:1	5.8	20
**Polyunsaturated FA**		**1.8**	**3**
	18:2	1.8	2.7
	18:3	0	0.3
**Cholesterol**		**0**	**0.2**

Oil composition was extracted from the USDA National Nutrient Database for Standard Reference. Values are in g/100 g.

**Table 3:** Lipids, protein and carbohydrate repartition from the standard, coconut and butter diet

**week**	**3**			**16**		
	
	**standard**	**coco**	**butter**	**standard**	**coco**	**butter**
	
**kcal/rat/day**	**45**	**64**	**62**	**73**	**178**	**95**
**% lipid**	**7**	**29**	**47**		**45**	**42**
% SFA	0.8	26.6	32.0		41.3	28.0
% MUFA	2.2	1.8	13.0		2.8	12.0
% PUFA	4.0	0.6	2.0		0.9	2.0
						
% from control diet		4	2		3	3
% from high fat diet		25	45		42	39
						
**% protein**	**16**	**14**	**13**		**13**	**13**
% from control diet		10	6		6	6
% from high fat diet		4	7		7	7
						
**% carbohydrate**	**77**	**57**	**40**		**42**	**45**
% from control diet		48	25		27	31
% from high fat diet		9	15		15	14

Compounds are expressed in % of total energy

An opposite picture was observed with MCD diet. Rats fed a MCD diet showed a smaller food intake (Fig. 1C) and a drop in body mass (Fig. 1D) as classically observed with this diet [30-32]. The mass of retroperitoneal white adipose tissue was dramatically reduced (-44%) in MCD rats (Fig. 2) which clearly indicates no fat accumulation in white adipose tissue. Conversely, the liver mass of MCD rat was higher than that of controls (4.5 ± 0.2 vs 3.4 ± 0.1 g/100g; P < 0.05).

### Liver histology and lipid content

**Figure 4:**
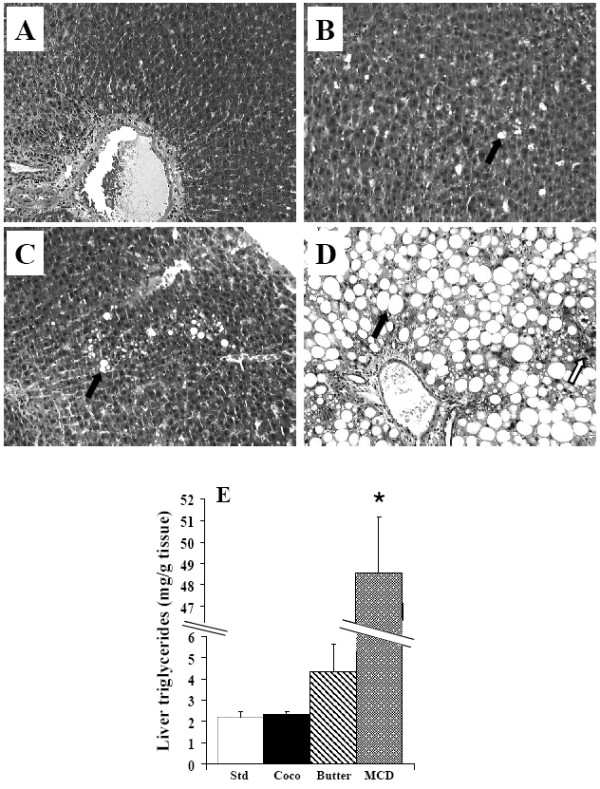
H&E and reticulin stained sections from rats fed standard diet (A), coconut diet (B), butter diet (C) for 14 weeks or MCD diet (D) for 6 weeks (magnification × 10), lipid vesicles are shown by a black arrow and inflammation point by a white arrow. (E) Liver triacylglycerol content (mg/g liver) in standard diet (std), coconut diet (Coco), butter diet (Butter) or MCD diet (MCDD). Results are means ± SEM, *P *< 0.05 vs standard.

Liver histology (Fig. 4A) from rats fed a standard diet showed no steatosis, inflammatory cells or fibrosis. The rats which were fed coconut oil or butter display a very mild macrovacuolar steatosis (affecting less than 5% of hepatocytes), whereas steatosis was severe (affecting more than 60% of hepatocytes) in the MCDD rats and associated to inflammation. This massive steatosis in the MCDD group was confirmed by triacylglycerol content, which was 22-fold higher in MCDD rats than in controls. High fat diet did not significantly increase liver triacylglycerol content (Fig. 4B). Thus, MCDD rats demonstrated the typical liver feature of non alcoholic steatohepatitis (steatosis and inflammation), whereas both high fat diets (coconut oil or butter) induced only very mild steatosis.

### Ability of the liver to oxidize fatty acids

**Figure 5:**
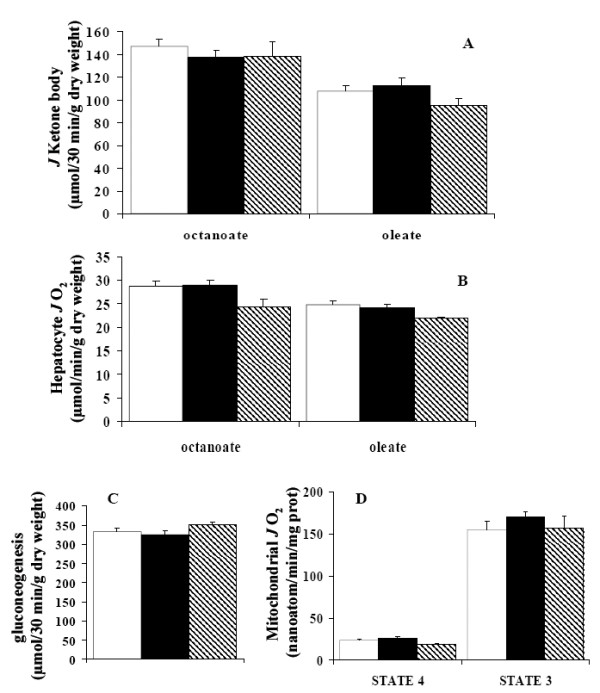
Liver capacity to oxidize fatty acids was assessed by (A) ketone body production and (B) oxygen uptake. Hepatocytes were isolated from 24 h starved Wistar rats. Cell viability was checked by a measure of (C) gluconeogenesis, in the presence of dihydroxyacetone and medium (octanoate) or long (oleate) chain fatty acids. (D) Rat liver mitochondria control state respiration was obtained with 5 Mm succinate/0.5 mM malate/1.25 μM rotenone as substrates. State 3 respirations were initiated with 1 mM ADP. Each determination was performed in triplicate from at least ten preparations for the three different diets: standard diet (white), coconut diet (black), butter diet (hatched). Results are means ± SEM. No significant difference between diets was observed.

In order to understand why rats fed a high fat diet did not develop liver injury, we searched for an increased capacity for fatty acid oxidation in liver cells. Fatty acid oxidation takes place in mitochondria and can be either partial, leading to ketone body synthesis (3-hydroxybutyrate and acetoacetate), or complete, leading to a large supply of reducing equivalents to the oxidative phosphorylation, thus resulting in a higher oxygen consumption. Fatty acid oxidation was therefore assessed by the measurement of both ketone body formation and hepatocyte respiration. We used either a long chain fatty acid (oleate), which is oxidized after transfer into the mitochondria by the carnitine-palmitoyl transferase (CPT), or a medium chain fatty acid (octanoate), that enters the mitochondria without any transporters. As expected, ketone body synthesis was higher with octanoate than with oleate, due to the direct entry of this fatty acid into the mitochondria. There was no significant change in ketone body formation in response to high fat diets, whatever the fatty acid used as substrate (Fig. 5A). This was corroborated by similar respiratory rate of hepatocytes from the three group studied (Fig. 5B).

Changes in dietary lipid can modify cell membrane composition that can, in turns, alter cell integrity resulting in a metabolism decrease. Hence, we studied gluconeogenesis which is controlled by, and therefore reflects, hepatocyte energy state. Using dihydroxyacetone as substrate, glucose production from isolated hepatocytes was not different in high fat diet groups as compared with standard group (Fig. 5C).

To verify that whole cell respiration measurement was not biased by the oxygen consumption of other organelles such as peroxisomes, the respiration capacities of isolated liver mitochondria were measured. Bovine serum albumin was added to eliminate any artefactual uncoupling effect of free fatty acids. No difference was found between mitochondrial oxygen consumption from high fat and standard diet fed rats (Fig. 5D). Therefore, resistance to steatosis was not explained by an enhanced ability of the liver to oxidize fatty acids.

### Brown adipose tissue, fatty acid oxidation and uncoupling protein 1

**Figure 6:**
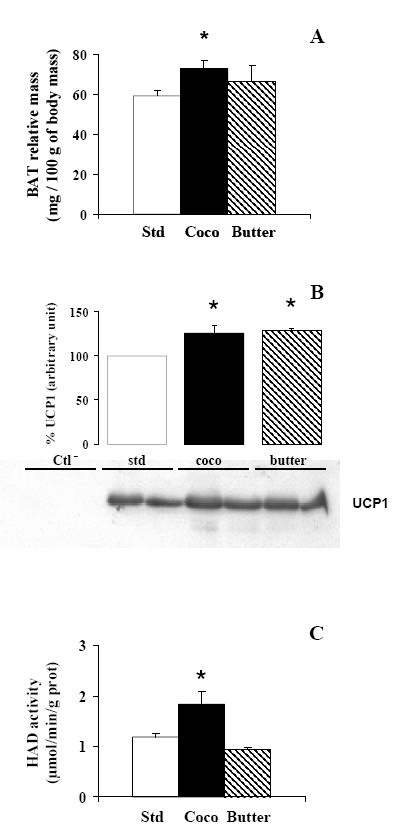
(A) Relative interscapular brown adipose tissue (BAT) mass in rats fed either a standard diet (Std), coconut diet (Coco) or butter diet (Butter). Data are means ± SEM; n = 10 for standard group, coco group, and n = 4 for butter group.  P < 0.05. (B) Western blot analysis of UCP1 content in brown adipose tissue from rat fed standard diet, coconut diet or butter diet. Detections were performed with 40 μg of BAT mitochondrial proteins. Negative control was liver from standard rats. (C) 3-hydroxyacyl-CoA dehydrogenase (HAD) activity is measured spectrophotometrically and expressed in micromoles per minute per gram protein. Data are means ± SEM; n = 8 for standard group, coco group, and n = 4 for butter group.  P < 0.05.

Despite enhanced lipid ingestion in the high fat diet animals, their body mass did not increase and fatty acids were not stored in the liver. Then, we searched for an other site of fatty acid utilisation and energy wasting. In rats, BAT is a well-known site for energy wastage due to the abundance of mitochondria and the profusion of an uncoupling protein (UCP1) in these mitochondria. Interscapular BAT depot was weighed and we estimated lipid oxidation by HAD activity and quantified the UCP1 protein content in high fat diet and standard diet fed rats. Intrascapular BAT depot was heavier (+23%) in coconut diet fed animals (Fig. 6A) while there was only a non significant trend in butter diet fed rat (+12%), possibly due to both little variations and a small number of rats in this group. A greater mitochondrial HAD activity was observed in coconut diet fed rats as compared with standard rats but not in butter fed rats (+55%; P < 0.05). A higher UCP1 protein content was observed in mitochondria from both coconut and butter diet fed rat (in arbitrary unit: coconut: +25%, butter: +28%; P < 0.05). Taken together, these results suggest a thermogenic activation of BAT that may result in fat oxidation contributing to the maintenance of body weight and the protection of liver.

## Discussion

In rodents, we succeeded in increasing lipid and caloric intake by a very large amount with an ad libitum access to diet. Nevertheless, this nutritional manipulation did not reproduce the typical hepatic lesion of NASH, i.e. steatosis, inflammation and fibrosis. No accumulation of triacylglycerols was observed in the liver of rats fed coconut oil containing 90% of SFA or butter with 51% of SFA. Such ability to overcome excessive energy intake may be related to rat ability to dissipate excess energy as heat. It is particularly true for young rats that resist becoming obese when fed a cafeteria-diet by increasing energy expenditure [33] through thermogenic processes occurring in liver [34] and BAT [35]. Our results show that, in rats fed a high fat diet, the ability of the liver to oxidize fatty acid, as assessed by i) ketone body formation, and ii) hepatocyte and mitochondrial respiration, is not enhanced. In our model, the liver of Wistar rats appears very mildly affected by an overload in lipid intake and we can assume that fatty acid exportation from the liver is sufficient to favour peripheral storage. High fat feeding probably induced an increased capacity to export triacylglycerol in the form of VLDL. Indeed it has been shown that feeding a diet with 20% hydrogenated coconut oil was shown to increase VLDL and LDL levels by 15–17% in rats and by 44% in mice. Furthermore plasma ApoE and ApoB were increased while hepatic ApoE mRNA and ApoB mRNA were unchanged [36]. Moreover, in our study, the high fat fed rats had increased white adipose tissue mass, which is in accordance with a higher triacylglycerol export from the liver to the adipose tissue. Similarly, feeding rats with various high fat diets (coconut oil, olive oil, menhaden oil, etc.) was shown to increase their epididymal fat mass [37-39]. However, we noted that fat accumulation in white adipose tissue was different depending on the type of fatty acid in the diets, and more precisely the length of the carbon chain. Indeed, coconut oil rich in medium chain saturated fat (mainly: lauric acid; C12:0; 44.6%) led to a lower peripheral accumulation than butter diet rich in longer chain saturated fat (mainly palmitic acid; C16:0; 21.7%). Many studies demonstrated that medium chain triglycerides (MCTs), such as in coconut oil, cause significant reduction in body weight or fat pad size in animals and humans [40-42]. This reduction of fat pad could be explained by the fact that MCT are transported directly to the liver *via *the portal vein and thus do not pass the adipose tissue before hepatic disposal. These characteristics could be responsible for the different rates of MCT oxidation versus LCT [43], and then could partly account for the difference in fat accumulation observed in white adipose tissue.

Another way to explain the lack of hepatic steatosis during lipid overload is peripheral utilisation. Mammals possess specialised thermogenic BAT that is characterized by a high amount of mitochondria containing high levels of UCP1, an uncoupling protein, located in the mitochondrial inner membrane [44]. UCP activation (by coldness or diet) results in the uncoupling of substrate oxidation from ADP phosphorylation [45], with a resultant increase in heat production [44]. In that thermogenic process, fatty acids act not solely as substrates for β-oxidation but are also involved in the uncoupling process by activating UCP1 transcription and activity [46]. UCP1 expression is regulated by a fatty acid activated transcriptional factor: peroxisome proliferator-activated receptor (PPAR) [47]. In our study interscapular BAT is larger, or tends to be larger, in the high fat fed groups, concomitantly with and increased content in UCP1, suggesting the implication of this tissue in fatty acid oxidation. BAT thermogenesis and UCP1 expression are known to increase during high-fat feeding, possibly to dissipate energy and to regulate body weight [35,47-49]. We can therefore postulate that rats can adapt to excessive lipid ingestion: firstly, by increasing the storage of fatty acids in peripheral white adipose tissues, and secondly by over-expressing the UCP1-related thermogenesis in BAT.

At this time, the reference model for the study of NASH is the MCD diet [31,32]. We confirm here that such diet induces a striking steatosis, demonstrated by a massive increase in hepatic triacylglycerol content. In the MCD diet-fed rats, steatohepatitis is the consequence of both the high-fat content and the methionine and choline deficiency. The lack of methionine reduces glutathione synthesis and impairs antioxidant defences against radical attacks. In addition, the choline deficiency impairs lipid exportation by decreasing the phosphatidylcholine synthesis, leading to a reduction in the fatty acid export from the liver [50]. The fact that, after an MCD diet, the high steatosis is associated with the blocking of the lipid export from the liver consorts with our hypothesis that, in our study, high-fat fed rats are resistant to liver injury thanks to a very efficient lipid exportation. Apart from steatohepatitis, the key feature of human NASH, the MCD diet fails to induce the other characteristics of NASH, i.e. abdominal obesity and increased calorie intake. Therefore, the MCDD model is adequate to study the consequence of fat accumulation and inflammation in hepatocytes but is inadequate to study the pathogenesis of steatohepatitis.

In this work, to mimic the diet habits of NASH patients [22], we realised a high fat diet with high level of medium chain SFA (i.e., coconut oil or butter). However, in rats, high fat diet with SFA (51% or 86%) was not efficient to induce steatosis or steatohepatitis. The comparison between the different high fat diet in Table 4[20,51-54], showed that there was a large variation in fat quantity in the regimens used in several studies. Surprisingly, it was not the diet with the higher percentage of fat that induced the most striking steatosis. The fattiest diets with 35–49% of lipid (w/w) (our model and the Lieber's or Zou's diets [20,53]) did not always induce steatosis or only a mild one (two fold increase as compared to their control). By contrast, a diet with "only" 10% of fat (w/w) developed steatosis and inflammation [54]. There is considerable evidence that the type and not the proportion of fat in a diet is a key determinant of fat accumulation and lesions in liver disease. Another interesting point was the percentage and the type of carbohydrates present in the diet. Indeed, increased dietary supply of carbohydrate could promote steatosis by increasing hepatic lipid uptake or *de novo *synthesis. Many studies showed that high sucrose supply induced obesity, insulino-resistance and steatosis in rodents [11,55-58]. Diets that were enriched with comparable amount of glucose or glycerol did not produce any over hepatic pathology. Surprisingly, in the studies presented in Table 4, steatosis was not always correlated with the presence of sucrose in the diet [20,59]. More studies are therefore needed to clarify the possible links between lipids and carbohydrates in NASH pathogenesis.

**Table 4:** Lipid characteristics of high fat diets used to induce steatosis and steatohepatitis

	**coconut diet**	**butter diet**	**Lieber**	**Zou**	**Rivera**	**Gauthier**		**Fan**
**Rat**	Wistar	Wistar	Sprague Dawley	Sprague Dawley	Wistar	Sprague Dawley		Sprague Dawley
**Fat (w/w)**	45% coconut	45% butter	30.1% corn oil 17.6% olive oil 1.7% safflower oil	35.7% corn oil	15% corn oil	3.4% corn oil 14.6% lard		10% lard oil 2% cholesterol
**SFA (% fat)**	86.5	51.3	13	13	13	34		39
**MUFA (% fat)**	5.8	21	41	24	27.6	42		45
**PUFA (% fat)**	1.8	3	42	59	54.7	20		21
**Carbohydrate (w/w)**	24.7% corn starch		16.2% dextrin maltose	13.4% sucrose	50% sucrose	39% corn starch		nd
**Time of diet (week)**	14	14	3	6	6	8	16	12
**steatosis**	no	no	mild	mild	no	yes	yes	yes
**inflammation**	no	no	yes	yes	no	nd	nd	mild
**fibrosis**	no	no	yes	nd	no	nd	nd	no

Abbreviations: SFA, saturated fatty acid; MUFA, monounsaturated fatty acid; PUFA, polyunsaturated fatty acid; nd, not determined.

The lipid composition of the different diets which induce steatohepatitis (see Table 4) [19,20,51,52,54], were lard and corn oil, both oils rich in unsaturated fatty acids. We can observe that fat of all the diets inducing steatosis and inflammation (Table 4) were richer in MUFA and PUFA (>30% and >20% of total fat respectively) as compared to our diet (5% and 2%). The injurious effect of unsaturated fatty acids, and particularly n-6 polyunsaturated fatty acids, was associated with enhanced lipid peroxidation and decreased concentrations of antioxidant enzymes, implicating oxidative stress as a causal factor. Indeed, different studies showed the pro-inflammatory effect of polyunsaturated n-6 fatty acids which exacerbate liver oxidative stress [60,61] and promote the development of NASH.

During the two last century, in Western diets, there has been a huge increase in n-6 fatty acid consumption and, the ratio of n-6 over n-3 fatty acids has increased from 1:1 to 15–20:1 [61,62]. Arachidonic acid (n-6) and eicosapentanoic acid (n-3) are precursors for the production of eicosanoids, and have opposite metabolic effects. Cardiovascular diseases, diabetes, obesity, cancer and other pathologies are associated with increased production of thromboxane A2, leukotriene B4, Il-1β, IL-6 and TNF. All these factors increase consequently to a rise in n-6 fatty acid intake and decrease with a higher n-3 fatty acid intake [63]. Different studies showed the pro-inflammatory effect of polyunsaturated n-6 fatty acids which exacerbate liver oxidative stress [63,64] and promote the development of NASH. It follows that the development of steatohepatitis with high fat diet in rats may be facilitated by the use of MUFA and PUFA, especially n-6 fatty acid, than SFA. However, these results contradict the observations made in humans where the daily intake of PUFA is around 5% (w/w) in the general population and 3.5% in NASH patients [22]. A high fat diet with saturated fatty acids is not sufficient to induce a steatosis and then a steatohepatitis. A number of studies showed, that it may be more suitable to use a high mono- and polyunsaturated fatty acid diet to induce NASH in rats. It appears here that the key factor could be the possible induction of the lipid peroxidation and pro-inflammatory cytokine production by the high level of PUFA leading to steatosis and inflammation. Another possibility is that Wistar rat is not a suitable model to study obesity and pathologic modifications in the liver consecutively to a modification of the diet [65].

## Conclusion

In conclusion, Wistar rats have an incredible capacity to adapt to a large increase of lipids in their alimentation. The mechanism underlying this resistance to high fat feeding is complex and involves both a change in body composition, with an increased storage in white adipose tissue, and an activation of lipid oxidation in BAT. We can conclude that, feeding Wistar rats with a high saturated fat diet does not induce liver failure and cannot be used as model of NASH. However, it is a good model for studying the adaptations of the organism to a high fat diet. Hence, it is still necessary to conceive a diet that can induce NASH, maybe by coupling a high fat diet with a stress, like inducing insulino-resistance or increasing ROS production.

## Competing interests

The author(s) declare that they have no competing interests.

## Authors' contributions

CR carried out the various experiments, participated in the design of the study and drafted the manuscript; CR, BS and EB performed animals and biochemical studies; VR, MD and IO performed histological analysis; CF and CD also helped in drafting manuscript; MAP and BS participated in the design of the study and helped to draft the manuscript. All authors read and approved the final manuscript.
